# Genomic heritability and correlation between carcass traits in Japanese Black cattle evaluated under different ceilings of relatedness among individuals

**DOI:** 10.3389/fgene.2023.1053291

**Published:** 2023-02-01

**Authors:** Elaheh Rostamzadeh Mahdabi, Rugang Tian, Yuan Li, Xiao Wang, Meng Zhao, Hui Li, Ding Yang, Hao Zhang, SuFan Li, Ali Esmailizadeh

**Affiliations:** ^1^ Department of Animal Science, Faculty of Agriculture, Shahid Bahonar University of Kerman, Kerman, Iran; ^2^ Institute of Animal Husbandry, Inner Mongolia Academy of Agricultural and Animal Husbandry Sciences, Hohhot, China

**Keywords:** genomic heritability, genomic correlation, Japanese Black cattle, degree of relationship., carcass traits

## Abstract

The investigation of carcass traits to produce meat with high efficiency has been in focus on Japanese Black cattle since 1972. To implement a successful breeding program in carcass production, a comprehensive understanding of genetic characteristics and relationships between the traits is of paramount importance. In this study, genomic heritability and genomic correlation between carcass traits, including carcass weight (CW), rib eye area (REA), rib thickness (RT), subcutaneous fat thickness (SFT), yield rate (YI), and beef marbling score (BMS) were estimated using the genomic data of 9,850 Japanese Black cattle (4,142 heifers and 5,708 steers). In addition, we investigated the effect of genetic relatedness degree on the estimation of genetic parameters of carcass traits in sub-populations created based on different GRM-cutoff values. Genome-based restricted maximum likelihood (GREML) analysis was applied to estimate genetic parameters. Using all animal data, the heritability values for carcass traits were estimated as moderate to relatively high magnitude, ranging from 0.338 to 0.509 with standard errors, ranging from 0.014 to 0.015. The genetic correlations were obtained low and negative between SFT and REA [−0.198 (0.034)] and between SFT and BMS [−0.096 (0.033)] traits, and high and negative between SFT and YI [−0.634 (0.022)]. REA trait was genetically highly correlated with YI and BMS [0.811 (0.012) and 0.625 (0.022), respectively]. In sub-populations created based on the genetic-relatedness ceiling, the heritability estimates ranged from 0.212 (0.131) to 0.647 (0.066). At the genetic-relatedness ceiling of 0.15, the correlation values between most traits with low genomic correlation were overestimated while the correlations between the traits with relatively moderate to high correlations, ranging from 0.380 to 0.811, were underestimated. The values were steady at the ceilings of 0.30–0.95 (sample size of 5,443–9,850) for most of the highly correlated traits. The results demonstrated that there is considerable genetic variation and also favorable genomic correlations between carcass traits. Therefore, the genetic improvement for the traits can be simultaneously attained through genomic selection. In addition, we observed that depending on the degree of relationship between individuals and sample size, the genomic heritability and correlation estimates for carcass traits may be different.

## Introduction

Japanese Black cattle (so-called Wagyu), due to its unique characteristic, is one of the most important and well-known native cattle in Japan. The excellent quality of its meat has been noticed worldwide. This breed has been having significant genetic improvements in carcass traits since 1972 by applying a two-stage selection (Oikawa et al., 2006). Various traits including meat quality and quantity, food efficiency and, reproduction, with the priority of improving meat quality, have been the target of the breeding program (Gotoh et al., 2014; Gotoh et al., 2018; Oikawa, 2018). Over the years, intramuscular fat (more than 30% of the muscle masses) and monounsaturated fatty acids content have significantly increased, leading to a delicious taste and tenderness of the meat (Zembayashi et al., 1995; Horii et al., 2009; Albrecht et al., 2011). Recent goals in Japanese Black cattle breeding programs have been focused also on additional traits of economic importance not strictly related to meat quality, including carcass-related traits (Gotoh et al., 2014).

Before undertaking a successful breeding program in carcass production, it is necessary to understand the genetic characteristics of carcass traits including the genetic correlation between these traits. Recent genetic studies have been estimating genetic parameters (such as variance components, heritability, and genetic correlation) for carcass traits (Sasaki et al., 2006; Peters et al., 2014), and this information was applied in the improvement of beef cattle (Caetano et al., 2013; Do et al., 2016). The results vary according to breed, sample size, data type (genomic or pedigree-based information), and the statistical model used to estimate genetic parameters (Laird and Lange, 2011; Conley et al., 2014; Koh et al., 2014; Peters et al., 2014; Evans et al., 2018; Naserkheil et al., 2021). The precise and accurate estimation of genetic correlation between traits is important, especially in a selection index, because the selection of a trait can directly affect the selection of another trait. Since carcass traits are genetically correlated with each other, this can indirectly affect selection programs. In Japanese Black cattle, the genetic correlation between carcass-related traits (carcass weight, rib eye area, rib thickness, yield rate, and beef marbling score) has mostly been estimated positive, except for subcutaneous fat thickness with beef yield and marble score traits (Oyama, 2011).

Heritability indicates a proportion of phenotypic diversity in the population that has a genetic origin and can be inherited to the next-generation. High heritability values are an indicator of a population’s ability to respond quickly to selection (Falconer and Mackay, 1996; Wada et al., 2008). Among different studies, the heritability values ranged from 0.07 to 0.80 for the carcass traits of beef cattle (Kim et al., 2010; Oyama, 2011; Takeda et al., 2017; Zoda et al., 2022). The estimation of heritability can be conducted using analyses of either pedigree or genome-wide data. Over the last decade, with the advancement of whole-genome sequencing technology, both the genetic relationships between individuals and genetic parameters are often estimated using genomic data (Stanton-Geddes et al., 2013; Ryu and Lee, 2014). The GCTA (Genome wide Complex Trait Analysis) software, which is used to compute the genetic (co) variance over the genome, first, the genetic relationship matrix (GRM) between individuals constructs and then, the variance attributable to all SNPs computes with a genomic-based restricted maximum likelihood (GREML) approach (Yang et al., 2011a). Yang et al. (2011a) suggested that applying a cutoff of 0.025 on GRM to exclude close relatives can be helpful to capture the genetic variation by all the typed SNPs over the genome.

This study aimed to estimate genomic heritability, correlation, and variance components for carcass-related traits using 9,850 Japanese beef cattle genotyped for genome-wide SNPs. In addition, to highlight the significance and effect of the relatedness degree in the calculation of genetic parameters in animal traits, and also following Yang et al. (2013) recommendation to investigate the effect of different cutoff values on the estimates, we created sub-populations based on different relationship degrees, and then the genetic parameters were calculated in sub-populations.

## Materials and methods

The data were collected in the progeny testing program of the Japanese Black cattle by the Livestock Improvement Association of Japan, Inc. (LIAJ). The set of data analyzed here is fully explained by Onogi et al. (2021). Briefly, a total of 9,850 animals (4,142 heifers and 5,708 steers) from 487 sires were used in this study. The animals were obtained from 65 herds slaughtered between 2012 to 2018 (the average ages at slaughter ranged from 28.6–31.6 months for heifers and 27.6–30.0 months for steers). Phenotypic records were related to six carcass traits of Japanese Black cattle, including carcass weight (CW, kg), rib eye area (REA, cm^2^), rib thickness (RT, cm), subcutaneous fat thickness (SFT, cm), yield rate (YI, %) and beef marbling score (BMS). All of the animals were genotyped using BovineLD Genotyping BeadChip (Illumina, CA, United States) and then the genotypes were imputed to the higher density of the Illumina BovineSNP50 BeadChips using 1,223 sires and 4 dams as a reference and Beagle v4.0 software (Browning and Browning, 2007). Data pre-processing was performed using PLINK v1.9 (Purcell et al., 2007) by filtering SNPs with a minor allele frequency of less than 5%, an HWE (Hardy-Weinberg equilibrium) test below the $10^{-3}$ , a call rate less than 90% and individuals with 10% missing genotypes. A total of 33,738 SNPs remained for the subsequent analyses.

### Genetic and phenotypic correlations

We used bivariate linear mixed models (Thompson, 1973) to evaluate (co) variance components, heritability, and phenotypic and genetic correlations between carcass traits.

The animal model of two traits or bivariate is written as:

$$\left[\begin{array}{l} y_{1}\\ y_{2} \end{array}\right]=\left[\begin{array}{l} X_{1}\;0\\ 0\;X_{2} \end{array}\right]\left[\begin{array}{l} b_{1}\\ b_{2} \end{array}\right]+\left[\begin{array}{l} Z_{1}\;0\\ 0\;Z_{2} \end{array}\right]\left[\begin{array}{l} u_{1}\\ u_{2} \end{array}\right]+\left[\begin{array}{l} e_{1}\\ e_{2} \end{array}\right]\;\left(bivariate\right)$$ (1)

Where $y_{1}\;and\;y_{2}$ denote the observations vectors corresponding to individuals for traits 1 and 2, $b_{1}\;and\;b_{2}$ are the fixed effects vectors for traits 1 and 2, $u_{1}\;and\;u_{2}$ are vectors of the additive genetic effects for traits 1 and 2, and $e_{1}\;and\;e_{2}$ are the vectors of the residual effects, for traits 1 and 2. **X** and **Z** are incidence matrices related to effects **b** and **u**, respectively.

The (co)variance matrix of random effects is of the form:

$$V\;\left[\begin{array}{c} \begin{array}{c} u_{1}\\ u_{2}\\ e_{1} \end{array}\\ e_{2} \end{array}\right]=\left[\begin{array}{c} G\sigma_{a_{1}}^{2}\\ \begin{array}{c} G\sigma_{a_{2_{a_{1}}}}^{2}\\ \begin{array}{c} 0\\ 0 \end{array} \end{array} \end{array}\begin{array}{c} \begin{array}{c} G\sigma_{a_{1_{a_{2}}}}^{2}\\ G\sigma_{a_{2}}^{2} \end{array}\\ \begin{array}{c} 0\\ 0 \end{array} \end{array}\begin{array}{c} \begin{array}{c} 0\\ 0 \end{array}\\ \begin{array}{c} I\sigma_{e_{1}}^{2}\\ I\sigma_{e_{2}e_{1}}^{2} \end{array} \end{array}\begin{array}{c} \begin{array}{c} 0\\ 0 \end{array}\\ \begin{array}{c} I\sigma_{e_{1}e_{2}}^{2}\\ I\sigma_{e_{2}}^{2} \end{array} \end{array}\right]$$ (2)

Where $\sigma_{a}^{2}$ and $\sigma_{e}^{2}$ are the variance components for each trait, $\sigma_{a_{1a_{2}}}^{2}$ , $\sigma_{e_{1}e_{2}}^{2}$ are genetic and residual covariance between two traits, respectively, **I** is the identity matrix, **G** is a genomic relationship matrix (GRM) provided from a set of SNPs. The genetic relationship matrix calculates and scores a value for each pair of individuals in a data set using the estimates of genomic sharing and comparing two genomes. GRM was provided by Onogi et al. (2021) (https://doi.org/10.5061/dryad.tdz08kpz4). We prepared GRM binary file using R v4.1.3 software for the subsequent analysis (Team, 2013). In addition, we applied the phenotypic values adjusted for the fixed effects.

The genomic-based restricted maximum-likelihood (GREML) approach implemented in the Genetic Complex Trait Analysis (GCTA) software was used to estimate the genetic parameters. In the current study, we created sub-populations by different genetic-relatedness ceilings. The ceilings are specified using GCTA v1.94.0 software (GRM-cutoff option) (Yang et al., 2011a). It maintains individuals with a maximum determined degree of relatedness. Different cut-off relatedness values (0.15, 0. 20, 0.25, 0.30, 0.35, 0.40, 0.45, 0.50, 0.55, 0.60, 0.65, 0.70, 0.75, 0.80, 0.85, 0.90, and 0.95) were considered. Then, heritability, variance components, and correlation between the traits were computed using data sets from both the whole population (without the GRM-cutoff option) and sub-populations (with the GRM-cutoff option).

### Heritability and variance components

GREML approach used for estimating heritability detects the proportion of the differences in phenotypes that is due to genetics, by modeling random effects. For example, a phenotype can be defined as follows:

y=Xb+g+e (3)

where **g** is a vector of the individual genetic effects distributed as $g\;∼\;Normal\;\left(0,A\sigma_{g}^{2}\right)$ , e is a vector of residual effects distributed as $e\;∼\;Normal\;\left(0,I\sigma_{e}^{2}\right)$ . Phenotypic variance is estimated using restricted maximum likelihood by considering the GRM or additive relationships (**A**) matrix in the model. Then, phenotypic variance can be written in the following equation:

$$V\left(y\right)=A\sigma_{g}^{2}+I\;\sigma_{e}^{2}$$ (4)

where **I** is an identity matrix, and $\sigma_{g}^{2}\;and\;\sigma_{e}^{2}$ refer to the proportion of variation explained by the SNPs and error variance, respectively. Therefore, the heritability value ( $h_{SNP}^{2}$ ) is obtained by dividing $\sigma_{g}^{2}$ by ( $\sigma_{g}^{2}+\sigma_{e}^{2})$ . More details about the algorithm used were provided by yang et al. (2011).

There are positive or negative correlations between many economically important traits, which can cause undesired changes in the target traits. Therefore, the estimation of the correlation between traits is of considerable importance.

The genetic correlation parameter was calculated as $r_{G}={cov}_{A12}/\sqrt{\sigma_{A1}^{2}\sigma_{A2}^{2}}$ where ${cov}_{A12}$ is genetic covariance, $\sigma_{A1}^{2}$ and $\sigma_{A2}^{2}$ are additive genetic variance for traits and 2.

Phenotypic correlation was computed using $r_{P}={cov}_{P12}/\sqrt{\sigma_{P1}^{2}\sigma_{P2}^{2}}$ where ${cov}_{P12}$ is phenotypic covariance, $\sigma_{P1}^{2}$ and $\sigma_{P2}^{2}$ are phenotypic variance for traits 1 and 2.

## Results and discussion

### Estimation of heritability and variance components using the whole data set

The phenotypic values associated with carcass traits are presented entirely by Onogi et al. (2021). As shown in Table 1, the average values for CW, RE, RT, BMS, and YI were larger than those reported by previous studies in Japanese Black cattle (Mukai et al., 1995; Sasaki et al., 2006; Zoda et al., 2022). In this study, the SFT value (2.89 and 2.49 cm) was smaller than the values observed by Mukai et al. (1995); Sasaki et al. (2006). Over the years, breeding programs to improve carcass traits have probably led to a decrease in subcutaneous fat thickness. This can be advantageous because the energy over the maintenance and growth requirements will be stored as fat within the muscle rather than subcutaneous fat. Intramuscular fat has more oleic acid and less stearic acid compared to subcutaneous fat, which has positive effects on the quality (juiciness and flavor) of beef; besides, it may have slight health implications for consumers of marbled beef. (Troy et al., 2016; Schumacher et al., 2022). According to Gotoh et al. (2018), Japanese Black cattle have 5%–39% more intramuscular fat than European breeds.

**TABLE 1:** The average values of studied phenotypes.

	CW (kg)	REA (cm^2^)	RT (cm)	SFT (cm)	YI (%)	BMS (1–12)
Heifer	443.7 (52.5)	58.5 (9.7)	7.92 (0.89)	2.89 (0.81)	74.2 (1.6)	6.6 (2.0)
Steer	489.4 (54.3)	60.08 (10.6)	8.03 (0.85)	2.49 (0.71)	74.3 (1.6)	6.8 (2.1)

Numbers in parenthesis are standard deviation (SD).

CW, carcass weight; REA, rib eye area; RT, rib thickness; SFT, subcutaneous fat thickness; YI, yield rate; BMS, beef marbling score.

According to Oyama (2011), to design a successful breeding program considering the genetic variation parameter can also play an effective role in predicting the expected genetic response. In the current study, the estimated genetic variance for carcass traits (Table 2) was comparable with the results of Mukai et al. (1995). They estimated the genetic variance for CW, REA, RT, SFT, YI, and BMS traits (590, 17.5, 0.248, 0.417, 0.842, and 0.242, respectively) using 8,329 records of Japanese Black cattle slaughtered with an average age of 28.5 months. Though the Japanese Black cattle have been under continuous selection for carcass traits, we found an increase in genetic variance for CW, REA, BMS, and YI traits. This could be because some of the carcass traits are genetically uncorrelated. Moreover, observed differences in our results could be due to various factors such as the differences in population characteristics (sample size, sex, and birth year of animals), fixed and random effects included in the statistical model, data used to calculate the relationship matrix (A) (genomic vs. pedigree information), etc.

**TABLE 2:** Heritabilities, and variance components calculated from bivariate analysis of six carcass traits of Japanese Black cattle.

Traits	V (G)	V (e)	V (*p*)	h^2^
CW	1114.94 (49.554)	1080.4 (20.960)	2195.34 (45.264)	0.51 (0.014)
REA	39.02 (1.935)	50.95 (0.955)	89.97 (0.775)	0.43 (0.015)
RT	0.211 (0.012)	0.413 (0.007)	0.624 (0.011)	0.338 (0.015)
SFT	0.246 (0.012)	0.274 (0.005)	0.520 (0.011)	0.473 (0.015)
YI	1.020 (0.050)	1.271 (0.024)	2.289 (0.046)	0.446 (0.015)
BMS	1.821 (0.085)	1.927 (0.037)	3.748 (0.077)	0.486 (0.014)

Numbers in parenthesis are standard errors (SE).

CW, carcass weight; REA, rib eye area; RT, rib thickness; SFT, subcutaneous fat thickness; YI, yield rate; BMS, beef marbling score.

In this study, the heritability values for carcass traits were observed moderate to relatively high. Heritability for CW, REA, SFT, RT, YI, and BMS was estimated at 0.509, 0.434, 0.473, 0.338, 0.446 and, 0.486, respectively. Due to the presence of considerable genetic variation for these traits, an effective genetic improvement can be achieved through the genomic selection of superior animals. The estimated heritability for CW agreed closely with around 0.50 reported by Ogawa et al. (2016) in Japanese Black cattle, which was estimated based on SNP markers with different densities. However, the estimated heritability by Ogawa et al. (2016) for the BMS trait was higher (0.60) than the estimates of our study. McEwin et al. (2018); Oyama (2011) reviewed the unweighted average of heritability for carcass traits in Japanese Black cattle, and presented values similar to the present estimates for CW (0.48), REA (0.46), RT (0.38), and YI (0.48), whereas those for BMS (0.55) and SFT (0.39) were higher and lower than those obtained here, respectively. For most carcass traits, our estimates were lower than the values reported in earlier studies (Mukai et al., 1995; Inoue et al., 2015; Takeda et al., 2017; Takeda et al., 2018; Zoda et al., 2022). The estimated heritability for CW was lower than the values reported by Takeda et al. (2017) (0.57), Uchida et al. (2001) (0.64), Hoque et al. (2009) (0.6), and Zoda et al. (2022) (0.8). These results were somewhat expected because the estimated heritability values based on genomic data or single nucleotide polymorphisms are significantly lower than those are calculated by the traditional pedigree method (Visscher et al., 2010; Yang et al., 2010). This is probably due to the rare causative variants in regions of low linkage disequilibrium that are not tagged by common SNPs (Yang et al., 2010; Visscher et al., 2012; Zuk et al., 2012; Wainschtein et al., 2021). However, It was expected if more SNPs were genotyped, greater SNP heritability values would be estimated (Visscher et al., 2010), in other words, there is a positive correlation between the number of intragenic SNPs genotyped in each chromosome and the estimation of variance components (Yang et al., 2011b).

In breeding programs and predicting response to selection, the accurate estimation of heritability and other genetic parameters is of particular importance (Falconer and Mackay, 1996). The parameters are estimated using either pedigree or genomic information (Khalilisamani et al., 2022). To date, the use of pedigree for estimating the genetic parameter of quantitative traits has been widely used in various studies (Sasaki et al., 2006; Hoque et al., 2009; Takeda et al., 2018). However, the accuracy and completeness of the population information are debatable, which may lead to a biased estimate of the genetic components (Wainschtein et al., 2021; Zoda et al., 2022; Špehar et al., 2022). The estimated heritability based on pedigree information, because of gene-environment interaction, may be overestimated (Yang et al., 2015; Zhu and Zhou, 2020). The use of a relatedness matrix constructed based on molecular markers to calculate genetic parameters rather than pedigree information might be relatively more accurate and robust (Veerkamp et al., 2011; Bérénos et al., 2014; Kim et al., 2015; Kruijer et al., 2015; Srivastava et al., 2019), Nevertheless, SNP heritability may be downwardly biased due to inaccurate pre-processing of sequencing data (Zhu and Zhou, 2020).

The heritability estimate for carcass traits has also been compared with that of other published estimates of beef cattle. Srivastava et al. (2019), using 7,991 genotyped and pedigreed Hanwoo beef cattle, calculated the genomic heritability values for CW, REA, SFT, and BMS, in turn, 0.39, 0.39,0.39, and 0.46, which were lower than those found here. In the other study conducted on 13,717 Hanwoo cattle, genomic heritability of SFT (0.24), CW (0.25), REA (0.24), and BMS (0.27) was reported lower than our estimates (De las Heras-Saldana et al., 2020). The heritability estimate for SFT, CW, and REA traits was higher than those (0.16, 0.25, and 0.29, respectively) represented by Gordo et al. (2016) using both the numerator (A) and genomic (G) relationship matrix in Nellore cattle.

Several studies have estimated the genetic parameters of carcass traits using pedigree-based information in different beef cattle which are comparable with our results. We obtained the heritability values for carcass weight higher than those reported in Hanwoo (Mehrban et al., 2017), Angus (Saatchi et al., 2011), Simmental, Charolais, and Limousin but close to the estimates in Hereford and Aberdeen Angus (Kause et al., 2015). Our heritability estimate for the rib eye area was similar to the value observed for Hanwoo (Mehrban et al., 2017; Mehrban et al., 2021), but higher than those reported for Nellore (Zuin et al., 2012) and Angus (Saatchi et al., 2011). Riley et al. (2002) found that the heritability value of subcutaneous fat thickness for Brahman (0.63) was higher than the estimate of 0.473 in this study but our finding was similar to the results of Mehrban et al. (2017); Mehrban et al. (2021) for Hanwoo beef cattle (0.50 and 0.49, respectively). Another study represented higher heritability for beef marbling score (0.61) than our study (Mehrban et al., 2021). Other reports by Saatchi et al. (2011) in Angus (0.32) and Riley et al. (2002) in Brahman (0.44) were lower heritability estimates than that presented here. Heritability is a parameter that is specific to population and traits; therefore, it is normal that the estimated values are diverse in different studies. Overall, various factors such as breed, environmental variation, number and slaughter age of animals, developmental stage of the carcass, herd management, data type (genomic or pedigree information), and the statistical model used for analysis, may be involved in the different estimations of genetic parameters.

In the present study, the standard errors of the heritability estimates for carcass traits, ranging from 0.014 to 0.015, (n = 9,850) (Table 2) were lower than those reported in the literature, so, our estimates of heritability might be more reliable. Several studies have reported a wide range of standard errors, between 0.019–0.17, for the heritability estimates based on pedigree information in carcass traits (Hoque et al., 2005; Hoque et al., 2006; Oikawa et al., 2006; Hoque et al., 2009; Do et al., 2016; Mehrban et al., 2017; Takeda et al., 2018; Zoda et al., 2022). Moreover, Srivastava et al. (2019) obtained a mean SE of 0.025 using 7,991 genotyped Hanwoo cattle which was higher than that observed in this study. In this study, the use of a large dataset of genotyped animals has possibly led to a low standard error estimate.

### Estimation of genetic and phenotypic correlations of carcass traits using all animals

The calculated values of genetic and phenotypic correlations between carcass traits are given in Table 3. The genomic and phenotypic correlation between carcass traits ranged from −0.634 to 0.811 and −0.544 to 0.812, respectively. Investigating genetic correlations between traits can help determine whether one or both correlated traits need to be included in the selection program.

**TABLE 3:** Phenotypic and genetic correlation between carcass traits of Japanese Black cattle.

rG	CW	REA	RT	SFT	YI	BMS
CW		0.460 (0.026)	0.593 (0.023)	0.188 (0.031)	0.053 (0.033)	0.201 (0.031)
REA	0.460 (0.009)		0.380 (0.032)	−0.198 (0.034)	0.811 (0.012)	0.625 (0.022)
RT	0.595 (0.008)	0.41 (0.009)		0.131 (0.037)	0.302 (0.034)	0.382 (0.032)
SFT	0.247 (0.010)	−0.084 (0.010)	0.141 (0.010)		−0.634 (0.021)	−0.096 (0.033)
YI	0.075 (0.010)	0.812 (0.006)	0.383 (0.009)	−0.544 (0.008)		0.578 (0.024)
BMS	0.202 (0.010)	0.543 (0.008)	0.306 (0.010)	−0.067 (0.010)	0.499 (0.009)	

Above and below diagonal denoted to genetic and phenotypic correlations, respectively.

Numbers in parenthesis are standard errors (SE).

CW, carcass weight; REA, rib eye area; RT, rib thickness; SFT, subcutaneous fat thickness; YI, yield rate; BMS, beef marbling score.

When comparing the results with the pedigree-based studies, we found that the estimated genomic correlation between CW and REA (0.46) was similar to the value reported in the reviewed study by Oyama (2011) (0.44) in Japanese Black cattle, but opposed to the values previously reported: 0.23 (Mukai et al., 1995), 0.37 (Hoque et al., 2006) in Japanese Black cattle, 0.39 in Brahman (Smith et al., 2007), 0.52 (Choi et al., 2015), 0.80 (Do et al., 2016), and 0.55 (Mehrban et al., 2021) in Hanwoo cattle. Genetic correlation between two traits is represented that they have a common genetic background, because of pleiotropic effects, in the expression of traits (Lynch and Walsh, 1998). Kim et al. (2011) revealed that individuals carrying the DVL1 gene (with genotype CC) have increased both REA and CW. A relatively high genetic correlation was observed between RT and CW (0.539) in our study, which was inconsistent with the results of Oyama (2011) (0.70), Mukai et al. (1995) (0.75), Hoque et al. (2006) (0.85) in Japanese Black cattle. The genetic correlations between RT and SFT, YI, REA, and BMS were found to be low to relatively moderate (0.131–0.382), being close to the reviewed values (0.29–0.43) by Oyama (2011), but disagree with the results of Mukai et al. (1995)(0.06–0.43) and Hoque et al. (2006) (0.17–0.34). The high value of the estimated correlation between YI and REA (0.81) was similar to the value (0.84) reported by Oyama (2011); Hoque et al. (2006) and that reported by Uchida et al. (2001) (0.86) in Japanese Black cattle. The high genetic correlation between the two traits represents that measuring one of these traits is sufficient in breeding programs, in other words, selection for increased rib eye area can indirectly lead to higher yield rates. We also observed a positive and high genetic correlation between YI and BMS (0.578) and a negative and high one between YI and SFT (−0.634), which were in accordance with the finding of Hoque et al. (2006) in Japanese Black cattle, but disagree with the results obtained by others (Mukai et al., 1995; Oyama, 2011). Given the high correlation (positive and negative) between YI with fat traits, simultaneous improvement of BMS and SFT could be achieved by selecting Japanese Black cattle for carcass yield. However, YI and CW showed a genetic correlation close to zero and were consistent with the values of the previously reported in Japanese Black cattle (Mukai et al., 1995; Oyama, 2011) except for the value obtained (−0.14) by Hoque et al. (2006). The genetic correlation between REA and BMS was highly positive, being 0.625 (0.022), which was similar to the estimate of 0.63 by Hoque et al. (2006). A moderate to high value has also been reported in previous studies in Japanese Black cattle [0.43 by Oyama (2011) and 0.72 by Hoque et al. (2005)]. This is probably one of the excellent characteristics of this breed that makes it different from other breeds of beef cattle. A large amount of marbling that is placed between the muscle fiber bundles and separates them probably increases the measurement of the rib eye area (McEwin, 2016). Selection for REA and the quantity of carcass muscle would positively be accompanied by increased marbling. Both REA and BMS are significant economic traits that directly affect carcass prices (Do et al., 2016). Moreover, the marbling trait is the most significant factor in determining quality-grade beef (Kim et al., 2010). In other breeds, the genetic correlation between these two traits is low and in some studies, it has been estimated negative (Gregory et al., 1995; Smith et al., 2007; Kim et al., 2010; Choi et al., 2015; Mehrban et al., 2021). In most of the previous studies, positive, but low to the relatively moderate genetic correlation between CW and BMS were reported in Japanese Black cattle (0.09, 0.15 and 0.36 by Hoque et al. (2006); Oyama (2011); Mukai et al. (1995), respectively) and Hanwoo cattle [0.16 by Mehrban et al. (2021), 0.17 by Naserkheil et al. (2021); Choi et al. (2015)]; some of these studies were consistent with our finding (0.20). Furthermore, The CW was positively correlated with SFT (0.188) and was similar to the estimated value of 0.21 by Hoque et al. (2006) in Japanese Black cattle and, the values of 0.17, 0.15, 0.18, and 0.16 in Hanwoo cattle by others (Kim et al., 2010; Do et al., 2016; Mehrban et al., 2021). The estimate of a low correlation between CW and SFT was biologically defensible; because fat accumulation takes place after the reduction of relative muscle growth (Berg and Butterfield, 1968). Lack of, or weak negative, genetic correlation was observed between SFT and BMS (−0.096), which was in agreement with previous studies in Japanese Black cattle (Mukai et al., 1995; Hoque et al., 2005; Hoque et al., 2006; Oikawa et al., 2006; Oyama, 2011). A low and close to zero value was also reported in Hanwoo and Brahman beef cattle (Smith et al., 2007; Kim et al., 2010; Choi et al., 2015; Mehrban et al., 2021; Naserkheil et al., 2021). The findings of Boito et al. (2017) suggested that SFT does not affect marbling and palatability. This weak genetic correlation can be considered an advantage in further improving the marbling trait without increasing subcutaneous fat in beef cattle. In other words, selection for increased marbling is independent of any possible increase in subcutaneous fat deposition. A weak negative correlation (−0.20) was observed between subcutaneous fat thickness and rib eye area (SFT, REA). This estimate was close to the report of −0.25 by Hoque et al. (2006) in Japanese Black cattle, by Naserkheil et al. (2021) in Hanwoo and, by Smith et al. (2007) in Brahman. However, it was also comparable to that reported by Oyama (2011) (0.02), and Mukai et al. (1995) (−0.33) in Japanese Black cattle and by Zuin et al. (2012) (0.15) in Nelore, by Choi et al. (2015) (−0.30) and Do et al. (2016) (−0.07) and Mehrban et al. (2021) (−0.11) in Hanwoo cattle.

In a study based on genomic data, the genetic correlations between CW with BMS (0.2), REA (0.46), and SFT (0.15), and also between SFT with BMS (0.01) and REA (−0.23) in Hanwoo population (Srivastava et al., 2019) were obtained similar to our work.

To estimate accurate genetic correlations, large sample sizes are required (Lynch and Walsh, 1998). The average standard error for genomic correlations of carcass traits using 9,850 records was 0.028, ranging from 0.012 to 0.037, which was smaller than those obtained by the multi-trait genomic model in 7,991 genotyped Hanwoo cattle (0.01–0.05) (Srivastava et al., 2019).

It should be noted that the comparison of the genetic parameters estimated based on GRM with based on pedigree information (A matrix) must be done with caution. The genomic relationship matrix contains both IBD (identity by decent) and IBS (identity by state) information where as numerator relationship matrix is only considered identity by decent.

### Sub-populations created based on the degree of relationship

**FIGURE 1:**
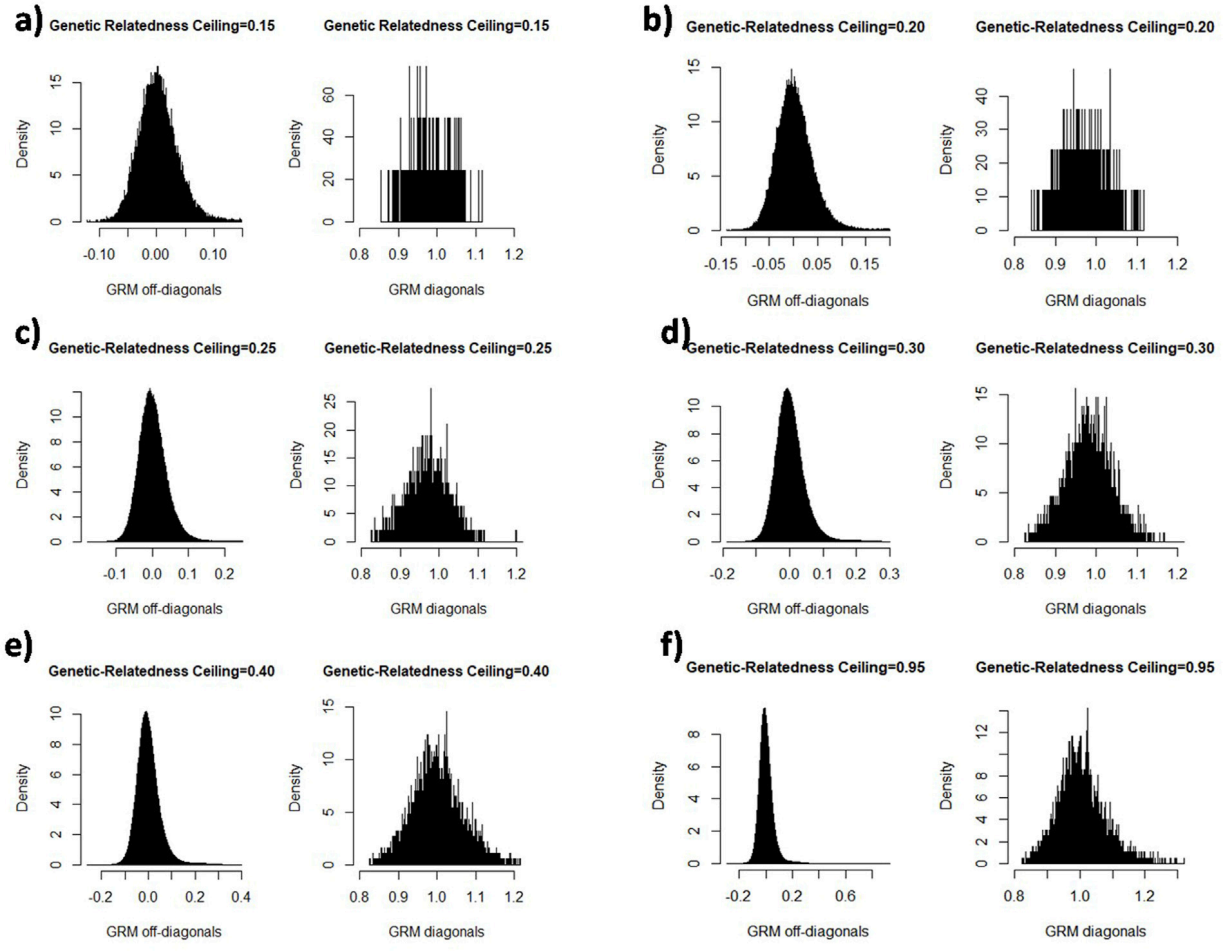
The illustration of the diagonal and off-diagonal elements of genomic relationship matrix in the cut-off relatedness different values: 0.15 **(a)**, 0.20 **(b)**, 0.25 **(c)**, 0.30 **(d)**, 0.40 **(e)**, and 0.95 **(f)**.

We created sub-populations depending on the degree of relationship among individuals. The diagonal and off-diagonal elements of the genomic relationship matrix are indicated in Figure 1. Each diagonal and off-diagonal element in the genomic relationship matrix is related to the self-relatedness and inbreeding coefficient of an animal and additive genetic relatedness between each pair of animals, respectively. At the cut-off of 0.15 and 0.20, off-diagonal elements had a higher density of negative estimates of relatedness that might be for two reasons: first, close relatives were excluded and more unrelated individuals were included in the sub-population, and second, the sample size was declined in the sub-population. Diagonal and off-diagonal elements indicated the same frequency and density at the relatedness ceilings of 0.35–0.95, in which subpopulations size were 8,180–9,850.

**FIGURE 2:**
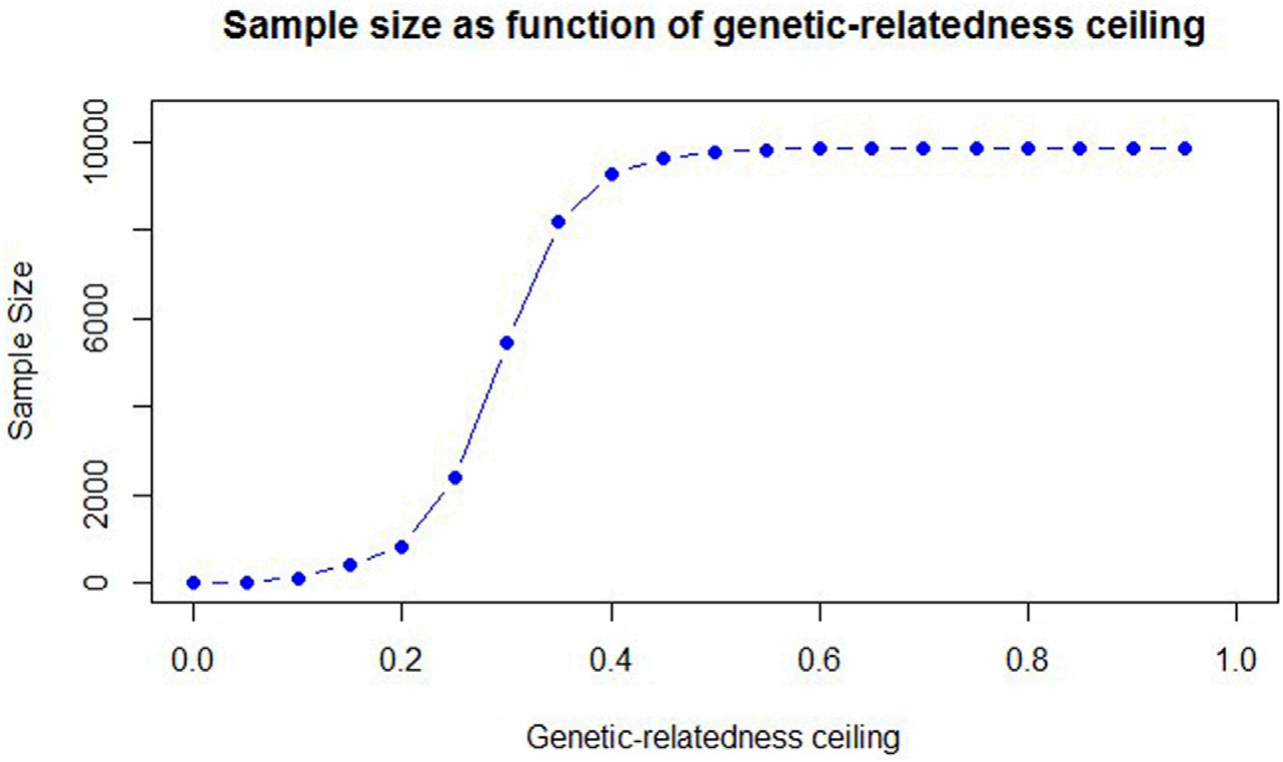
The change of sample size by applying the degrees of genetic-relatedness ceiling.

The size of the population varied under the defined ceilings. The sample size in the subpopulations is a consequence of applying the different degrees of relatedness to the main population and, it was not directly manipulated. According to Figure 2, the sample size increased with a steeper slope when the genetic-relatedness ceiling was between 0.2–0.4. With the increase of the relatedness ceiling, the participants increased, or in other words, from about the ceiling of 0.4 onwards, the number of eliminated individuals decreased. Below a ceiling of 15%, the number of animals was insufficient for the next analysis (less than 408). Therefore, the minimum and maximum relatedness ceiling were adjusted to 0.15 and 0.95, respectively.

### Estimation of heritability of carcass traits using sub-populations

**FIGURE 3:**
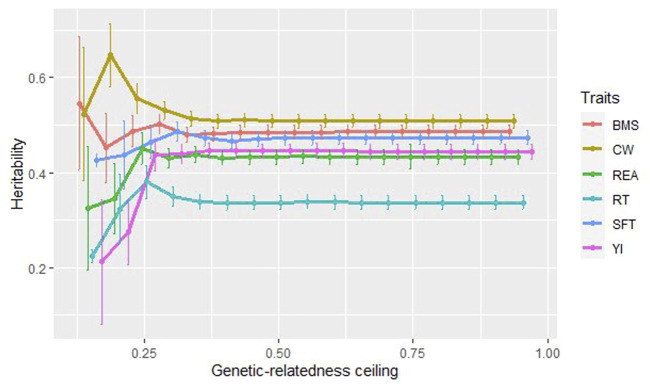
The estimated heritability for carcass traits at the different degrees of genetic-relatedness ceiling.

Figure 3 depicts the heritability values for carcass traits at 17 sub-populations created based on the degree of genetic relationship. At the ceiling of 0.15–0.30 for all carcass traits, the heritability values fluctuated, especially for carcass weight and the beef marbling score. A noticeable increase and decrease in the heritability values were observed around the ceiling of 0.20 (sample size = 833) for CW (0.647, SE = 0.066) and BMS (0.452, SE = 0.073), respectively. The lowest heritability for all traits was estimated at 0.15 ceiling (sample size = 408), except for the beef marbling score (0.545, SE = 0.14). It was in agreement with Kirkpatrick et al. (2014), who reported the lowest ${\hat{h}}_{SNP}^{2}$ values at the ceiling <0.15. For REA and RT traits, the highest heritability value was related to the ceiling of 0.25 (sample size = 2,370).

At genetic-relatedness ceilings of 0.30–0.95, when the population size was between 8,182 and 9,850, the heritability values were steady at about 0.508 for CW, 0.433 for REA, 0.337 for RT, 0.474 for SFT, 0.444 for YI, 0.486 for BMS. Traits with low to moderate heritability (RT, REA, and YI) were more affected by decreasing population size and indicated more fluctuations of estimates at low degrees of relationship. It has been shown that as the genetic-relatedness ceiling increases, the precision of heritability estimates increases (Kirkpatrick et al., 2014). In our study, from the ceilings of 0.40 (sample size = 9,285) onwards, the standard error was evaluated at 0.015 for all traits. It can be due to an increase in population size in higher genetic-relatedness ceilings and as a result, accuracy is increased (Kirkpatrick et al., 2014).

### Estimation of heritability of carcass traits using sub-populations

Figure 3 depicts the heritability values for carcass traits at 17 sub-populations created based on the degree of genetic relationship. At the ceiling of 0.15–0.30 for all carcass traits, the heritability values fluctuated, especially for carcass weight and the beef marbling score. A noticeable increase and decrease in the heritability values were observed around the ceiling of 0.20 (sample size = 833) for CW (0.647, SE = 0.066) and BMS (0.452, SE = 0.073), respectively. The lowest heritability for all traits was estimated at 0.15 ceiling (sample size = 408), except for the beef marbling score (0.545, SE = 0.14). It was in agreement with Kirkpatrick et al. (2014), who reported the lowest ${\hat{h}}_{SNP}^{2}$ values at the ceiling <0.15. For REA and RT traits, the highest heritability value was related to the ceiling of 0.25 (sample size = 2,370). At genetic-relatedness ceilings of 0.30–0.95, when the population size was between 8,182 and 9,850, the heritability values were steady at about 0.508 for CW, 0.433 for REA, 0.337 for RT, 0.474 for SFT, 0.444 for YI, 0.486 for BMS. Traits with low to moderate heritability (RT, REA, and YI) were more affected by decreasing population size and indicated more fluctuations of estimates at low degrees of relationship. It has been shown that as the genetic-relatedness ceiling increases, the precision of heritability estimates increases (Kirkpatrick et al., 2014). In our study, from the ceilings of 0.40 (sample size = 9,285) onwards, the standard error was evaluated at 0.015 for all traits. It can be due to an increase in population size in higher genetic-relatedness ceilings and as a result, accuracy is increased (Kirkpatrick et al., 2014).

### Estimation of genetic correlations of carcass traits using sub-populations

**FIGURE 4:**
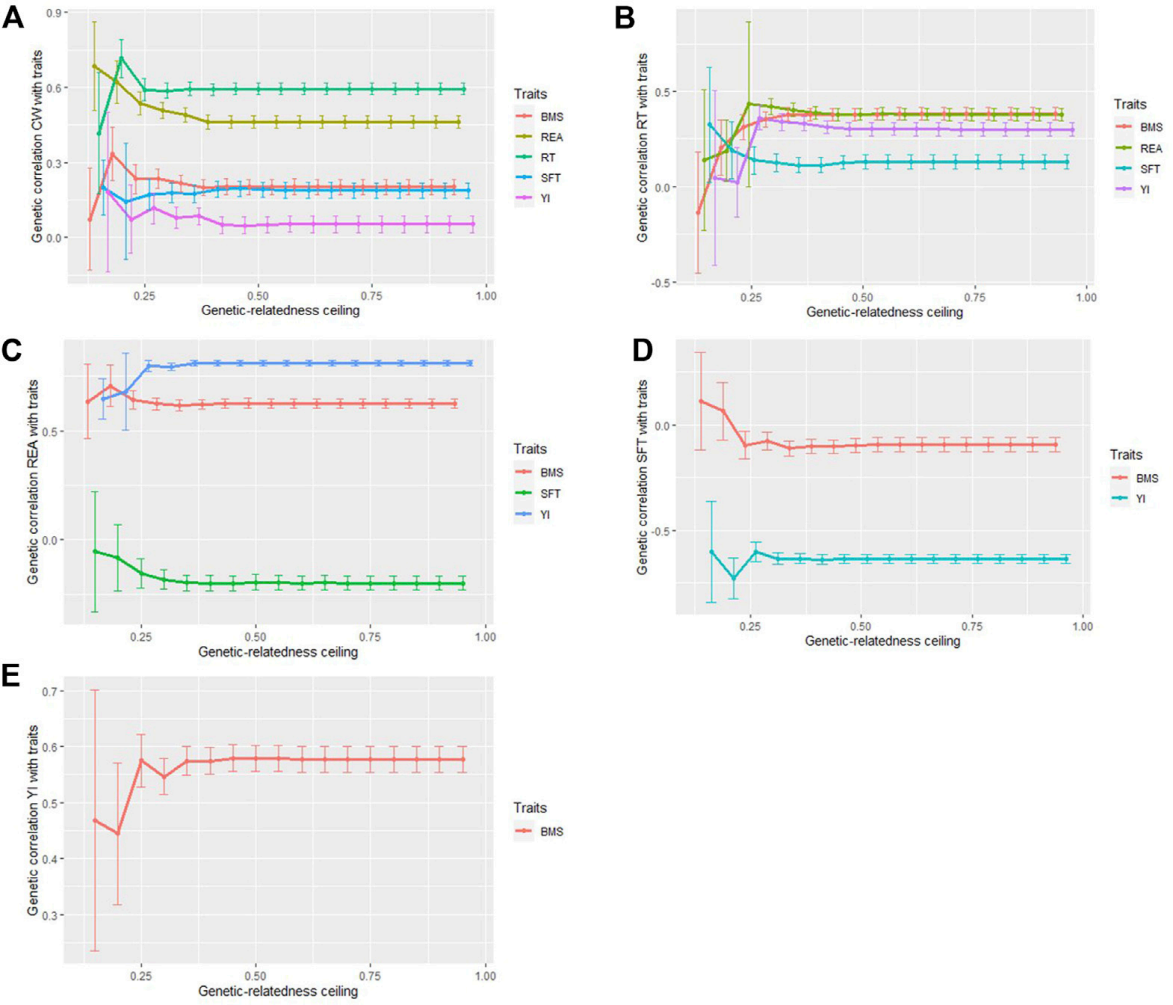
Genetic correlations between carcass traits in different genetic-relatedness ceilings. Genetic correlation between CW and REA, RT, SFT, YI, BMS **(A)**, RT and REA, SFT, YI, BMS **(B)**, REA and SFT, YI, BMS **(C)**, SFT and YI, BMS **(D)**, YI and BMS. **(E)**.

Both sample size and the degree of relationship between individuals affect the estimation of genomic correlations between traits (Figure 4). At the ceiling of 0.15 (sample size = 408), traits with low genomic correlation, including CW and YI (0.053), REA and SFT (−0.198), SFT and BMS (0.096), CW and SFT (0.188), and RT and SFT (0.131), was overestimated except for CW and BMS. Genetic correlation between traits with moderate and high correlation, ranging from 0.380 to 0.811, except for CW and REA, was underestimated in the sub-populations with smaller sample sizes (sample size = 408). Between ceilings of 0.25–0.95, the estimates were steady in most traits with high correlation, whereas the estimates for traits with low genetic correlation were relatively stable from the ceilings of 0.40 onwards.

In a study, Yang et al. (2011a) reported that the estimates of heritability would be more like the obtained estimates from a pedigree analysis if there are a remarkable proportion of closely related individuals in a family study because the estimates could be confounded with some non-additive and non-genetic effects shared between closely related individuals (Yang et al., 2013). They recommended a relationship ceiling of 0.025 in such populations. Kim et al. (2015) indicated that the degree of genetic relationship between individuals can be an effective and important factor in estimating heritability. In beef cattle populations, due to breeding programs such as selection and controlled mating to achieve higher production efficiency, individuals with close kinship relationships may be included in a herd, and as a result, the close relatives (parent-offspring pairs and sibling) may cause a biased estimate of genetic variance attributed to all the SNPs (Yang et al., 2011a). In the current study, we compared the estimate of genetic parameters in sub-population (beef cattle belonging to 65 herds) with different cutoff values to understand whether the estimates of genetic parameters change when closely related animals remained in the population. At the genetic-relatedness ceiling <0.25 (for example, when half-siblings are present in the data), with a sample size of 2,370 (about 24% of the total population), all carcass traits showed fluctuations in the estimated genetic parameters in different ways. These differences in estimates compared to when is applied any GRM-cutoff, could be also due to the population size. At a cutoff of 0.30 with a sample size of 5,443 (about 83% of the total population), the results of the estimates were close to the estimates obtained from the genetic relatedness ceiling of 0.95, in other words, with the addition of about 1,670 individuals with a degree of kinship >0.30, the estimate of genetic parameters for most traits was similar to the results of the full data analysis without GRM-cutoff. Therefore, if there is a large population of genotyped animals with a genetic relationship less than 0.30, the presence of individuals with higher kinship may not affect the estimations in comparison with when the GRM-cutoff value is not applied. Overall, our estimates were affected by two sources of variation (the degree of genetic relatedness and sample size) because the sample size was a consequence of the specified cut-off value for relationships between individuals so population sizes varied at different genetic relatedness ceilings. It should be noted that if sufficient data are available, we can apply a range of relatedness ceilings with the same population sizes to account for the changes based only on genetic-relatedness among individuals.

## Conclusion

Our results indicated that there is moderate to relatively high heritability for carcass traits, therefore genomic selection can be effective to improve these traits in Japanese Black cattle. The highest and lowest heritability estimates were related to carcass weight and rib thickness, respectively. A favorable genetic correlation was observed between beef marble score with subcutaneous fat thickness and rib thickness. This is a unique characteristic that distinguishes Japanese Black cattle from other beef cattle. In addition, we revealed that the values of genomic heritability and correlation between carcass traits may be differently estimated, depending on sample size and correlation between individuals. Compared to the estimates obtained from the analysis of the full data without GRM-cutoff, the lowest heritability values were obtained at the subpopulations with low relationship degree and sample size, for most carcass traits; moreover, traits with relatively moderate to high genetic correlation were underestimated at the low genetic-relatedness ceilings.

## Data Availability

The datasets presented in this study can be found in Dryad (https://doi.org/10.5061/dryad.tdz08kpz4).
